# Operative Results of Early Surgical Treatment

**Published:** 1918-08

**Authors:** Pierre Duval, E. Vaucher


**Operative Results of Early Surgical Treatment of Wounds of the Lung.** By PIERRE DUVAL, Consulting Surgeon of the 7th Army, and E. VAUCHER, Bacteriologist, Autochir. 21.

For the last two years Dr. Duval has treated the wounds of the lungs presenting serious immediate accidents, external or internal bleeding, and opened thorax, by immediate surgical operation on the wound of the lung. For the past year and a half he has operated systematically without compulsion of urgency on certain wounds of the chest to remove the foreign bodies (missile, splinters), to clean surgically the wound of the lung (excision, sweeping suture), to clean the pleura and to treat the wound of the chest wall[1].

The speaker wants to show today the general results of this treatment, the functional results demonstrated by radiological examination of the wounds thus operated.

Without any immediate surgical treatment the total mortality of chest wounds reaches 30 o/o, not including the soldiers who die in the midposts of the regiments :

| | |
|---|---|
| Through and through wounds . . . . . . . . . . | 21.2 o/o |
| Wounds with shell fragment retained. . . . . | 30.3 " |
| Wounds with opened thorax. . . . . . . . . . | 27 " |
| Wounds with closed thorax . . . . . . . . . . | 15 " |

These figures are based on 3,453 cases.

The authors' personal statistics of last year including all cases from the field ambulance to the evacuation hospital, all the cases who died without having been operated, those operated on for serious bleeding or opened thorax, those operated systematically, and those not operated, are taken from 161 cases among which there were 27 deaths, a mortality of 16.7 per cent.

1. For the reasons for this treatment, the indications for operating, and the technic, see PIERRE DUVAL, *Les plaies de guerre du poumon*. English translation by Colonel Thompson. Masson et C^ie^, éditeurs, Paris, 1917.

Of these 27 soldiers who died, 13 were not operated. They died soon after reaching the ambulance in such bad state that nothing could be done to save them. 148 cases could be treated. 14 died. Mortality 10.5 o/o.

Among these 148 cases, 29 were operated on through urgency, either because they were bleeding severely — 16 cases, 9 died — or because the thorax was opened — 13 cases, 4 died — the mortality among these being 13.

The mortality among the cases operated on through urgency was 44.8 per cent.

There were 119 cases which probably would have been treated medically in other ambulances.

102 were treated medically because there was no indication for operating; 5 developed empyema; 1 of them healed perfectly; 1 died of pleural infection — it was the only death among the 102 cases; the 17 remaining cases which presented no urgent indication for operation but which, according to the speaker's personal experience, would probably have developed infection, were nevertheless operated on immediately after reaching the ambulance: the indications for operating being given either by the size of the missile or the injury of the chest wall, fracture of ribs or of 'scapula, or the importance of the hematoma in the lung. This prophylactic operation consists in:

Removal of the foreign body;

Direct treatment of the wound of the lung;

Bleeding of the pleural cavity;

Careful excision of the wound of the chest wall, which has given the following results.

Death 0.

12 Complete healings with

6 perfect results,

6 very good results.

5 cases with complications after operation:

3 localized empyema containing anaerobic bacilli,

1 empyema of the whole pleural cavity containing streptococci,

1 abscess of the lung.

These 5 cases healed well after a secondary operation had been performed.

The speaker's opinion was that these infections were due to the fact that the wound of the lung had not been thoroughly treated, incomplete excision, bits of clothing, or splinters remaining in the wound or in the pleura, and the missile not removed.

All operated cases were looked after clinically every day, repeated exploratory punctures were done to test the fluid bacteriologically. Every four or five days, more often if necessary, a radioscopical examination was done at the bed of the patient with the mobile X ray.

In most cases the pneumothorax had disappeared after five or six days and the lung was in close connection with the chest wall. There remained during an average of 15 to 30 days a little shadow diffused through the whole lung or localized in one or two places often in the lower part of the pleura.

If there was some fluid, it often disappeared in eight to ten days. We evacuated the liquid as soon as possible when necessary. In most cases thus treated, the chest was normal, on an average, in from 15 to 30 days — the dilation of the thorax normal, the movement of the diaphragm completely free. In 6 cases there remained a slight general opacity of the wounded side, due probably to thickening of the pleura. The authors have not been able to follow their cases long enough to determine how long this opacity persists.

If there is infection, it is habitually a mild infection; there are one or two loculated pockets in the pleura; serious generalized infection of the whole pleural cavity should be exceptional.

The general impression of such cases is that the quality of the healing is much better than when primitive operation has not been performed.

*Conclusions.*

Systematic primitive operation on the wound of the lung gives general results much superior to those afforded by medical treatment. The statistics established on 3453 cases treated medically with surgical treatment of the complications give :

| | |
|---|---|
| Mortality | 30 o/o |
| Pleural infection | 25 " |
| Mortality of pleural infections | 40 " |

The authors' statistics of last summer including all the cases from the first field ambulance to the Evacuation Hospital, based on 161 cases give :

| | |
|---|---|
| Mortality | 16.7 o/o |
| Pleural infection | 15 " |
| Mortality of pleural infection | 20 " |

They believe that, if the indications for operating established in their previous publications are carefully followed, and if the prophylactic operation is done promptly, during the first twelve or twenty-four hours, the bad prognosis of the wounds of the lung will be considerably diminished.

Systematic surgical treatment is the logical and real prophylactic treatment of the infection of the pleura and of the lung. The quality of healing obtained by this method of procedure seems much better than that which is obtained by medical treatment.

